# Reference Intervals for Serum Cystatin C and Factors Influencing Cystatin C Levels Other than Renal Function in the Elderly

**DOI:** 10.1371/journal.pone.0086066

**Published:** 2014-01-21

**Authors:** Lu Wei, Xiaoshuang Ye, Xiaohua Pei, Jianqing Wu, Weihong Zhao

**Affiliations:** Department of Geriatrics, the First Affiliated Hospital of Nanjing Medical University, Nanjing, Jiangsu, China; University of São Paulo School of Medicine, Brazil

## Abstract

**Objective:**

The present study aimed to establish reference intervals for serum cystatin C (Scys-C) stratified by stages of chronic kidney disease, explore factors influencing Scys-C and compare the performance of Scys-C with serum creatinine (Scr) in the young and elderly.

**Methods:**

A total of 800 participants, 516 young (<60 years) and 284 old (≥60 years) subjects were included in this study. Scys-C and Scr were assayed by the partical-enhanced immunoturbidimetry method and enzymatic method respectively. 95% reference interval was adopted to evaluate reference intervals. Influencing factors were characterized by multivariate linear regression analysis. Relationship between reference glomerular filtration rate (rGFR) and Scys-C or Scr was determined by correlation coefficient.

**Results:**

Reference intervals for Scys-C were calculated to be 0.71–1.38 mg/L, 0.83–1.67 mg/L, 1.02–2.61 mg/L, 1.32–4.48 mg/L, 1.95–6.11 mg/L in the aged in CKD G1, G2, G3a, G3b and G4-5 stages, respectively. Body mass index(BMI), nephritis, kidney neoplasm and hypertension were demonstrated as factors affecting Scys-C in the elderly while gender, nephritis and kidney neoplasm were clarified as influencing factors in the young group. Scr levels were affected by more factors, such as body surface area and hematological disease. Correlation coefficient between rGFR and Scys-C or Scr showed that serum Scys-C was superior to Scr, especially in the subjects with mildly decreased renal function (−0.593 vs. −0.520).

**Conclusions:**

Factors other than renal function influenced Scys-C when applying to evaluate glomerular filtration rate (GFR), such as BMI, nephritis, kidney neoplasm and hypertension, and Scys-C had higher correlation with GFR than Scr in the elderly.

## Introduction

Chronic kidney disease (CKD) and end-stage renal disease (ESRD) in particular are major health problems worldwide with dramatically increasing incidence and prevalence [1]. Several studies were conducted among the elderly and showed a markedly high prevalence [2]. In China, the incidence of CKD is 10.8%, that is to say, there are at least 100 million patients with CKD [3]. The evaluation of glomerular filtration rate (GFR) is very important to diagnosis of CKD. Thus, assessing GFR accurately in early stage of CKD is essential for clinician to achieve explicit diagnosis and take reasonable therapies.

As we know, GFR can be determined by measuring the clearance of exogenous substances or evaluated by the serum or urinary concentration of endogenous substances. Serum creatinine (Scr) has been used to assess renal function as a traditional endogenous substance for many years, but several factors other than renal function have been certified to affect Scr level, for instance muscle mass, age, gender and malnutrition etc [4]. Inulin clearance or nuclear medicine techniques such as 99Tc DTPA or 51Cr EDTA, which is considered as the golden standard measurement of GFR, is expensive, cumbersome and invasive [5]. Thus, a new, convenient and relatively accurate endogenous substance is needed to evaluate renal function for clinical application.

Serum cystatin C (Scys-C) recently was proposed as a promising alternative marker of GFR owing to better specificity and sensitivity for detecting mildly decreased GFR. However, several studies have reported that Scys-C was superior to Scr as a marker of GFR [18], [19], in contrast, some studies didn't show this advantage [4], [20]. The existence of factors other than renal function influencing Scys-C may lead to this discrepancy especially in the elderly, although these factors are not completely clear. In addition, aging has becoming a serious social problem worldwide [6], and failed physiological functions and pathologic abnormalities in the elderly probably lead to the different performance of Scr and Scys-C in evaluation of renal function between the young and elderly. But, few studies focused on the comparison between these two groups. Thus, further detailed studies are needed to evaluate the performance of Scys-C compared with Scr, especially in the elderly.

Therefore, the present study aimed to establish reference intervals for Scys-C in subjects stratified by age and stages of CKD and identified factors other than renal function influencing Scys-C. Besides, the performance of Scys-C and Scr as a GFR marker was compared in subjects stratified by age and early or advanced stage of CKD.

## Methods

### Subjects and measurements

Totally 800 participants, 516 young (age<60 ys) and 284 old subjects (age≥60 ys), who were outpatients or inpatients of our hospital from December 2009 to March 2013 were included. The basic therapies of these participants were anti-hypertensive drugs, oral hypoglycemic drugs, corticosteroid etc. Reference glomerular filtration rate (rGFR) was measured by the 99mTc-DTPA renal dynamic imaging on a single photon emission computed tomography (Siemens E.CAM, Siemens Co., Ltd, Germany) [7], and Scys-C concentration was assayed by the partical-enhanced immunoturbidimetry method (Beijing Leadman Biomedical Co., Ltd, China) with a reference range of 0.60–1.55 mg/L. Meanwhile, Scr levels were analyzed using enzymatic method on Shanghai kehua Dongling Diagnostic Products with a reference range of 44–136 µmol/L. Both two markers were examined by an Olympus AU5400 autoanalyzer (Olympus Co., Ltd, Japan). All participants provided their written informed consent to participate in this study and Nanjing Medical University Ethics committee approved this study.

### Reference intervals

Included subjects were divided into ten groups stratified by age (<60 ys and ≥60 ys) and stages of CKD according to decreased degrees of GFR (G1, G2, G3a, G3b and G4–5 stages). According to the KDIGO clinical practice guideline for evaluation and management of CKD, it further acknowledges the importance of dividing stage 3 based on data supporting different outcomes and risk profiles in categories G3a (GFR 45–59 ml/min/1.73 m^2^) and G3b (GFR 30–44 ml/min/1.73 m^2^) [8], [9]. Thus, we divided the stage 3 into G3a and G3b stages to calculate reference intervals separately. Since Scys-C concentrations were not normally distributed, the natural logarithm of Scys-C value which was normally distributed after transformed was used for this analysis. Reference intervals for Scys-C were calculated by 95% reference interval. Single-factor ANOVA analysis was used to determine the statistical significance of differences between groups stratified by CKD stages. Simultaneously, the difference between the young and elderly was evaluated using t-test.

### Influencing factors and correlation coefficients

Multivariate linear regression analysis was performed to analyze influencing factors other than renal function on Scys-C concentrations. Scys-C and rGFR levels were ln-transformed. BMI, body surface area (BSA) and age were expressed as the initial data. Gender, nephritis, kidney neoplasm, hematological disease, hypertension and diabetes mellitus (DM) were used as binary factors. The same analysis was performed on Scr.

Correlation coefficients were assessed using Spearman's rank correlation. We calculated R values between rGFR and Scys-C or Scr in early stages and advanced stages respectively.

### Statistical analysis

Statistical analysis was performed using SPSS 17.0 for Windows. Data was presented as median. Mann-Whitney-Wilcoxon test and Chi-square test were adopted to determine the statistical difference between the young and elderly. Two-tailed *P* value of <0.05 was considered to be statistically significant.

## Results

Characteristics of the included subjects were presented in Table 1. The rGFR of the elderly was significantly lower than that of the young, whereas, age, BMI, and the levels of Scys-C and Scr in the elderly were higher than those in the young subjects. The totally constituent ratio of hematological disease, hypertension, coronary heart disease and DM was statistically different between the young and elderly (Table 2), however, the constituent ratio of all basic diseases had no age-related significant difference in the G1 and G2 stages.

**Table 1:** Characteristics of the study subjects.

	<60 ys	≥60 ys	Total
N (%)	516(64.5)	284(35.5)	800
Women (%)	208(40.3)	107(37.7)	315(39.4)
Age (ys)	45(33–53)	69(64–75)**	53.5(41–65)
BMI (kg/m^2^)	22.49(21.48–24.22)	22.49(21.48–24.91)**	22.49(21.48–24.48)
BSA (m^2^)	1.73(1.56–1.78)	1.75(1.60–1.79)	1.74(1.57–1.79)
Serum cystatin C (mg/L)	0.98(0.84–1.19)	1.34(1.11–1.95)**	1.09(0.91–1.41)
Serum creatinine (µmol/L)	74.9(59.63–93.65)	97.3(77.93–134.1)**	82.2(65.6–106.58)
rGFR (ml/min/1.73 m^2^)	86.05(71.43–101.95)	62.8(46.08–74.85)**	76.95(59.73–93.7)

BMI: Body mass index, BSA: Body surface area, rGFR: Reference glomerular filtration rate measured by the 99mTc-DTPA renal dynamic imaging.

** *P*<0.01 compared with the young.

**Table 2:** Basic diseases of included subjects.

	<60y	≥60y	Total
	N (%)	N (%)	N (%)
Nephritis	28(5.4)	18(6.3)	46(5.8)
Kidney neoplasm	132(25.6)	89(31.3)	221(27.6)
Hematological disease	101(19.6)	10(3.5)**	111(13.9)
Hypertension	70(13.6)	92(32.4)**	162(20.3)
Coronary heart disease	7(1.4)	28(9.9)**	35(4.4)
Diabetic mellitus	26(5.0)	58(20.4)**	84(10.5)

** *P*<0.01 compared with the young.

According to age and stages of CKD, all subjects were divided to ten groups, and the corresponding reference intervals, means and medians of Scys-C of each group were shown in Table 3. Reference intervals for Scys-C were calculated to be 0.56-1.27 mg/L, 0.72–1.51 mg/L, 0.98–2.61 mg/L, 1.57–4.01 mg/L, 1.80–10.49 mg/L in the young group and 0.71–1.38 mg/L, 0.83–1.67 mg/L, 1.02–2.61 mg/L, 1.32–4.48 mg/L, 1.95–6.11 mg/L in the elderly group in G1, G2, G3a, G3b and G4-5 stages, respectively. Scys-C levels of the elderly were significantly higher than those of the young with normal renal function (*P* = 0.001), while the rGFR values were not significantly different between above-mentioned two groups (*P* = 0.121), which suggested that age was a factor influencing Scys-C in the patients with normal GFR. The one-way ANOVA analysis and pairwise comparisons revealed statistically significant difference of Scys-C between G1, G2, G3a, G3b and G4-5 stages in both two groups, thus to the clinician, Scys-C could be used to evaluate the decreased degree of GFR.

**Table 3:** Reference intervals of serum cystatin C in young and old subjects.

	<60 y				≥60 y			
	n	Reference interval	Mean	Standard deviation	n	Reference interval	Mean	Standard deviation
G1	218	0.56–1.27	0.84	0.18	20	0.71–1.38	0.99*	0.17
G2	223	0.72–1.51	1.04	0.21	136	0.83–1.67	1.18*	0.22
G3a	45	0.98–2.61	1.59	0.44	61	1.02–2.61	1.64	0.43
G3b	22	1.57–4.01	2.50	0.60	42	1.32–4.48	2.43	0.87
G4-5	8	1.80–10.49	4.76	1.51	25	1.95–6.11	3.59	0.84

* *P*<0.05 compared with the young.

Table 4 showed the results of multivariate linear regression analysis in all, the young and the elderly subjects. In terms of all included subjects, gender, nephritis and kidney neoplasm were significantly associated with serum concentrations of Scys-C besides the age (*r* = −0.062, 0.155 and −0.072, respectively). In the young group, nephritis was independently positively associated with Scys-C concentrations (*r* = 0.146) while the female gender and patients with kidney neoplasm had lower Scys-C concentrations after adjustment for rGFR (*r* = −0.094 and −0.063, respectively), whereas, BMI, hematological disease, hypertension and DM did not have significantly correlation with Scys-C (*P*≥0.05). Compared with the young group, Scys-C was influenced by more factors in the elderly. After adjustment for rGFR, kidney neoplasm were negatively associated with Scys-C (*r* = −0.064) while BMI, nephritis and hypertension were all significantly positively associated with it (*r* = 0.020, 0.138 and 0.064, respectively), and gender, hematological disease and DM did not have effect on Scys-C in the elderly(*P*≥0.05). As expected, Scr levels were influenced by more factors, such as BSA, hematological disease and the influencing power of the same factors was stronger than Scys-C after adjustment for rGFR.

**Table 4:** Influencing coefficients of factors on Scys-C and Scr.

Variable	<60y		≥60y		Total	
	Scr	*Scys-C*	Scr	*Scys-C*	Scr	*Scys-C*
Adjusted for rGFR						
Gender	−0.263^**^	−0.094^**^	−0.096	0.079	−0.182^**^	−0.062^**^
BSA	−0.123	−0.135	3.968*	0.429	0.333^**^	−0.003
BMI	−0.002	0.005	0.133*	0.020^**^	0.016^**^	0.002
Nephritis	0.224^**^	0.146^**^	0.176^**^	0.138*	0.206^**^	0.155^**^
Kidney neoplasm	−0.081^**^	−0.063^**^	−0.086*	−0.064*	−0.084^**^	−0.072^**^
Hematological disease	−0.248^**^	−0.013	−0.163*	0.081	−0.243^**^	0.006
Hypertension	0.051	0.005	0.081*	0.064*	0.061^**^	0.036
Diabetic mellitus	−0.026	0.075	0.015	0.014	0.005	0.034

BMI, Body mass index, BSA, Body surface area, rGFR, Reference glomerular filtration rate, Scys-C, serum cystatin C, Scr, serum creatinine.

* *P*<0.05, ^**^ *P*<0.01.

**Figure 1:**
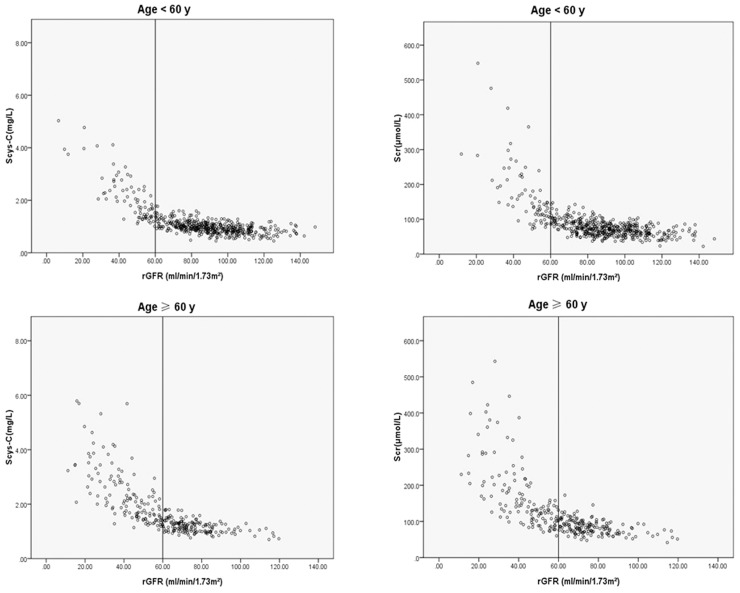
Relationship between rGFR and serum CysC or Scr stratified by age and progression of CKD (Scys-C, serum cystatin C, Scr, serum creatinine, rGFR, reference glomerular filtration rate).

The correlation coefficients of Scys-C were consistently higher than those of Scr in subjects stratified by age, and the young had better correlation with rGFR than the elderly especially in the early stage of CKD, but there was not significant difference in correlation coefficients between Scys-C and Scr. Negative relationship between rGFR and the Scys-C or Scr was shown and Scys-C had greater correlation and accuracy than Scr (Table 5 and Figure 1).

**Table 5:** Correlation coefficients of Scys-C or Scr and rGFR.

		CysC	Scr
Total	rGFR≥60 ml/min/1.73 m^2^	−0.593	−0.520
	rGFR<60 ml/min/1.73 m^2^	−0.743	−0.699
<60 y	rGFR≥60 ml/min/1.73 m^2^	−0.523	−0.462
	rGFR<60 ml/min/1.73 m^2^	−0.746	−0.715
≥60 y	rGFR≥60 ml/min/1.73 m^2^	−0.423	−0.421
	rGFR<60 ml/min/1.73 m^2^	−0.732	−0.719

rGFR, Reference glomerular filtration rate, Scys-C, serum cystatin C, Scr, serum creatinine.

## Discussion

Scr has been widely used to assess renal function for a while though it can not accurately predict renal function because of its poor sensitivity, especially in the early stage of CKD. Recently, several studies have clarified that Scys-C is superior to Scr in evaluation of GFR, thus Scys-C was proposed as an alternative parameter to evaluate renal function. But, few studies concentrated on reference intervals for Scys-C to evaluate progression of CKD and factors other than renal function influencing Scys-C, and fewer studies explored the difference between the young and elderly. Therefore, the main objectives of our study were to establish reference intervals for Scys-C and analyze factors other than renal function influencing Scys-C.

Reference intervals were established by 95% reference interval using the natural logarithm of Scys-C values and difference among groups was evaluated by t-test and one-way ANOVA analysis. In the early stage, defined as rGFR≥60 ml/min/1.73 m^2^, reference intervals of the elderly were significantly higher than those of the young, but no significant difference of rGFR levels and the constituent ratio of all basic diseases were found in G1 stage between the two groups. Hence, age may be a factor influencing Scys-C levels in patients with normal renal function. Besides, the force of age would decrease along with the reduced GFR, then decreased renal function would be the main influencing factor taking place of age when GFR decreased below 60 ml/min/1.73 m^2^. The one-way ANOVA analyses was adopted to examine the difference between five stage groups, and the results showed significant difference. These results may provide a reference to clinician that Scys-C can be used as a parameter to assess the progression of CKD, and provide evidence that the elderly have higher Scys-C levels than the young when the renal function is normal or mildly-decreased.

Some studies have investigated that many factors other than renal function may influence Scys-C, and more studies are needed for verification [10], [11], [12]. Multivariate linear regression analysis was conducted in three groups, all subjects, the young and the elderly. Female gender was associated with lower Scys-C in young subjects (*P*<0.001), but in the elderly, it was not an influencing factor (*P* = 0.836). Some studies revealed that Scys-C was influenced by gender, while Yashiro et al. reported that Scys-C levels were not gender-related [10], [11], [12]. Decreased secretion of sex hormone in the elderly may contribute the difference between these two groups. Stevens et al. reported that percent change of Scys-C was higher than the value of Scr according to the change in BMI (mean 27.7 kg/m^2^), and speculated an association between fat mass and Scys-C [13]. But, in this study, BMI (mean 22.84 kg/m^2^) was not associated with Scys-C in the young group, similarly with the results examined in patients from Japan, with a mean BMI of 22.9 kg/m^2^ [11]. Meanwhile, the mean of BMI in the elderly was 23.47 kg/m^2^ and was significantly different from this of the young group, which may explain statistically significant effect of BMI on Scys-C in the old group. Race and the prevalence of obesity may contribute to the inconsistent conclusions regarding the association with BMI.

In both young and elderly groups, kidney neoplasm was negatively associated with Scys-C concentrations. A review showed that tumors have been suggested to influence Scys-C production, although this is still widely debated [14]. In the present study, included subjects with kidney neoplasia may have a relatively increased renal function, which result in the negatively association between kidney neoplasm and Scys-C. Hence, more studies are needed to clarify whether kidney neoplasm is an influencing factor on Scys-C.

Nephritis acted as a positively correlative factor in both two groups. The application of corticosteroid, which is essential drug in the treatment of some kinds of nephritis may influence Scys-C concentrations. The hypothesis that increased Scys-C was associated with corticosteroid doses had been demonstrated in asthmatics, adult renal transplant patients, and patients with nephritis [12], [14]. Besides, the patients with nephritis were in the inflammatory status, and inflammation acted as an influencing factor has been clarified by many studies, which found that Scys-C was associated with many inflammatory markers like IL-6, TNF-α but not with C-reactive protein (CRP) [15].

Hypertension, as a common chronic disease and classical cardiovascular risk factor in the elderly, was positively associated with Scys-C concentrations in the elderly. Some studies have reported that the prevalence of an elevated Scys-C in the general population was found to be high and was associated with the presence of classical cardiovascular risk factors such as DM, hypertension [12], [16], [17]. But, DM was not an influencing factor in our study. This converse results may due to the diabetes sample size and its low constituent ratio, and LA Stevens et al. demonstrated that the association of higher Scys-C with diabetes may, in part, also reflect the association with fat mass, which inferred that low fat mass of included subjects may be another reason leading to this variance [13]. Given the high and increasing prevalence of hypertension and DM, these are important consideration to clinician when using Scys-C to evaluate renal function, especially in the elderly.

Compared with Scys-C, Scr levels were affected by more factors other than renal function, such as BSA, hematological disease. Besides, the influencing power of the same factors was stronger on Scr, especially in the elderly. This result suggested that considering to the influencing factors other than renal function, Scys-C was better than Scr in the evaluation of renal function.

The superior of Scys-C was more than in the terms of less influencing factors, and the advantage on the correlation with rGFR was clarified by correlation coefficient. We calculated the correlation coefficient in early and advanced stages of CKD. The performance of Scys-C was surpassing than Scr in both the young and the elderly. But the advantage was not apparent in the old subjects with normal to mildly-scathing renal function, and correlation between Scys-C and rGFR of the old subjects was worse than that of the young subjects though statistically significant difference was not detected in both groups. Some factors such as age and chronic underlying diseases which are incidental in the elderly may result in this disadvantage. The majority of compositions demonstrated that Scys-C was superior than Scr in terms of correlation coefficient with rGFR while little of these demonstrated if statistically significant difference existed, but a part of those got opposite results [4], [18], [19], [20].

Factors other than renal function influenced Scys-C when applying to evaluate glomerular filtration rate, such as BMI, nephritis, kidney neoplasm and hypertension, and Scys-C had higher correlation with GFR than Scr in the elderly. But, the difference was existed between the young and the elderly, so clinician needs to consider the difference and the influencing factors when applying Scys-C to evaluate renal function in the elderly. In addition, the influencing factors also should be considered when using Scys-C to creat equations to estimate GFR.

## References

[1] Stefan HR, Arend B, Walter H (2007) How to estimate GFR-serum creatinine, serum cystatin C or equations? Clin Biochem 40: 153–161. 17234172 10.1016/j.clinbiochem.2006.10.014

[2] Zhang QL, Dietrich R (2008) Prevalence of chronic kidney disease in population-based studies: Systematic review. BMC Public Health 8: 117 10.1186/1471-2458-8-117 PMC2377260 18405348

[3] Zhang L, Wang F, Wang L, Wang WK, Liu BC, et al (2012) Prevalence of chronic kidney disease in China: a cross-sectional survey. Lancet 379: 815–822. 22386035 10.1016/S0140-6736(12)60033-6

[4] Vikas RD, Charles K, Gary S (2002) Serum Cystatin C Is Superior to Serum Creatinine as a Marker of Kidney Function: A Meta-Analysis. Am J Kidney Dis 40: 221–226. 12148093 10.1053/ajkd.2002.34487

[5] Jung YC, Sung HP, Seong KK (2010) Serum Cystatin C is a Potential Endogenous Marker for the Estimation of Renal Function in Male Gout Patients with Renal Impairment. J Korean Med Sci 25: 42–8. 20052346 10.3346/jkms.2010.25.1.42 PMC2800003

[6] Moye J, Marson DC, Edelstein B (2013) Assessment of capacity in an aging society. Am Psychol 68: 158–71. 23586491 10.1037/a0032159 PMC4160021

[7] Pei X, Yang WY, Wang SN, Zhu B, Wu JQ, et al (2013) Using Mathematical Algorithms to Modify Glomerular Filtration Rate Estimation Equations. PloS One 8: e57852. 23472113 10.1371/journal.pone.0057852 PMC3589471

[8] KDIGO Clinical Practice Guideline for Evaluation and Management of CKD (2012) KDIGO Public Review Draft. http://kdigo.org/home/

[9] Go AS, Chertow GM, Fan D, McCulloch CE, Hsu CY (2004) Chronic kidney disease and the risks of death, cardiovascular events, and hospitalization. N Engl J Med 351: 1296–1305. 15385656 10.1056/NEJMoa041031

[10] Knight EL, Verhave JC, Spiegelman D, Hillege HL, de Zeeuw D, et al (2004) Factors influencing serum cystatin C levels other than renal function and the impact on renal function measurement. Kidney International 65: 1416–1421. 15086483 10.1111/j.1523-1755.2004.00517.x

[11] Masaru H, Enyu I, Yoshinari Y, Tsuyoshi W, Seiichi M (2011) Performance of serum cystatin C versus serum creatinine as a marker of glomerular filtration rate as measured by inulin renal clearance. Clin Exp Nephrol 15: 868–876. 21861242 10.1007/s10157-011-0525-y

[12] Masatomo Y, Tadashi K, Hiroyoshi S, Yuko K, Toru M, Eri M (2009) Comparisons of cystatin C with creatinine for evaluation of renal function in chronic kidney disease. Clin Exp Nephrol 13: 598–604. 19585181 10.1007/s10157-009-0202-6

[13] Stevens LA, Schmid CH, Greene T, Li L, Beck GJ, et al (2009) Factors other than glomerular filtration rate affect serum cystatin C levels. Kidney International 75: 652–660. 19119287 10.1038/ki.2008.638 PMC4557800

[14] Sophie SV, Pierre D, Laurence P, Christophe M, Marc F, et al (2008) Cystatin C: current position and future prospects. Clin Chem Lab Med 46(12): 1664–1686. 18973461 10.1515/CCLM.2008.336

[15] Okura T, Jotoku M, Irita J, Enomoto D, Nagao T, et al (2010) Association between cystatin C and inflammation in patients with essential hypertension. Clin Exp Nephrol 14(6): 584–8. 20809110 10.1007/s10157-010-0334-8

[16] Anoop S, Srinivas T (2011) Relationship between serum cystatin C and hypertension among US adults without clinically recognized chronic kidney disease. J Am Soc Hypertens 5(5): 378–384. 21498146 10.1016/j.jash.2011.03.003 PMC3140570

[17] Cepeda J, Tranche-Iparraguirre S, Marín-Iranzo R, Fernández-Rodríguez E, Riesgo-García A, et al (2010) Cystatin C and cardiovascular risk in the general population. Rev Esp Cardiol 63(4): 415–22. 20334807

[18] Pei XH, He J, Liu Q, Zhu B, Bao LH, et al (2012) Evaluation of serum creatinine- and cystatin C-based equations for the estimation of glomerular fi ltration rate in a Chinese population. Scand J Urol Nephrol 46(3): 223–31. 22376289 10.3109/00365599.2012.660985

[19] Jeon YK, Kim MR, Huh JE, Mok JY, Song SH, et al (2011) Cystatin C as an Early Biomarker of Nephropathy in Patients with Type 2 Diabetes. J Korean Med Sci 26(2): 258–63. 21286018 10.3346/jkms.2011.26.2.258 PMC3031011

[20] Herget RS, Trabold S, Huesing J, Heemann U, Philipp T, et al (2000) Cystatin C-An accurate marker of glomerular filtration rate after renal transplantation? Transpl Int 13: 285–289. 10959481 10.1007/s001470050703

